# Evaluation of Skeletal and Cardiac Muscle Function after Chronic Administration of Thymosin β-4 in the Dystrophin Deficient Mouse

**DOI:** 10.1371/journal.pone.0008976

**Published:** 2010-01-29

**Authors:** Christopher F. Spurney, Hee-Jae Cha, Arpana Sali, Gouri S. Pandey, Emidio Pistilli, Alfredo D. Guerron, Heather Gordish-Dressman, Eric P. Hoffman, Kanneboyina Nagaraju

**Affiliations:** 1 Division of Cardiology, Children's National Medical Center, Washington, District of Columbia, United States of America; 2 Department of Parasitology and Genetics, Kosin University College of Medicine, Amnam-dong, Seo-gu, Busan, South Korea; 3 Center for Genetic Medicine Research, Children's National Medical Center, Washington, District of Columbia, United States of America; 4 Pennsylvania Muscle Institute, School of Medicine, University of Pennsylvania, Philadelphia, Pennsylvania, United States of America; Hospital Vall d'Hebron, Spain

## Abstract

Thymosin beta-4 (Tβ4) is a ubiquitous protein with many properties relating to cell proliferation and differentiation that promotes wound healing and modulates inflammatory mediators. We studied the effects of chronic administration of Tβ4 on the skeletal and cardiac muscle of dystrophin deficient *mdx* mice, the mouse model of Duchenne muscular dystrophy. Female wild type (C57BL10/ScSnJ) and *mdx* mice, 8–10 weeks old, were treated with 150 µg of Tβ4 twice a week for 6 months. To promote muscle pathology, mice were exercised for 30 minutes twice a week. Skeletal and cardiac muscle function were assessed via grip strength and high frequency echocardiography. Localization of Tβ4 and amount of fibrosis were quantified using immunohistochemistry and Gomori's tri-chrome staining, respectively. *Mdx* mice treated with Tβ4 showed a significant increase in skeletal muscle regenerating fibers compared to untreated *mdx* mice. Tβ4 stained exclusively in the regenerating fibers of *mdx* mice. Although untreated *mdx* mice had significantly decreased skeletal muscle strength compared to untreated wild type, there were no significant improvements in *mdx* mice after treatment. Systolic cardiac function, measured as percent shortening fraction, was decreased in untreated *mdx* mice compared to untreated wild type and there was no significant difference after treatment in *mdx* mice. Skeletal and cardiac muscle fibrosis were also significantly increased in untreated *mdx* mice compared to wild type, but there was no significant improvement in treated *mdx* mice. In exercised dystrophin deficient mice, chronic administration of Tβ4 increased the number of regenerating fibers in skeletal muscle and could have a potential role in treatment of skeletal muscle disease in Duchenne muscular dystrophy.

## Introduction

Duchenne muscular dystrophy (DMD) is an inherited X-linked disorder with an incidence of 1 in 3,500 male births that is due to the absence of dystrophin, a large protein linking the intracellular cytoskeleton to the extracellular matrix.[1] The animal model of DMD, the *mdx* mouse, is genetically similar to the human deletion.[1]–[3] Although the underlying gene defect is the same in human and the *mdx* mouse, the clinical picture is quite different. The *mdx* skeletal muscle undergoes an early acute phase of degeneration at 3–4 weeks of age followed by a successful regeneration phase. The histopathology after this acute phase shows a relatively mild picture, although specific muscles (e.g. diaphragm) and older mice can show more severe pathology consistent with human DMD muscle at presentation (failed regeneration and fibrosis). Commensurate with the pathology, the physical symptoms of the *mdx* mouse tend to be relatively mild, with muscle weakness more obvious after exercise or lengthening contractions.[4]–[6] *Mdx* mice also develop decreased cardiac systolic function slowly over time. This decreased function can be measured at significant levels by non-invasive echocardiography around nine months of age.[7], [8]

In order to identify new potential therapeutic agents, studies have looked at skeletal muscle gene expression profiles in *mdx* mice during disease progression. [9]–[17] Thymosin beta-4 (Tβ4) was one gene with increased expression in dystrophin deficient skeletal muscle cells and may play a role in compensatory pathways.[11], [16]–[18] Tβ4 is a peptide of 43 amino acids that was first isolated from the thymus gland and subsequently found to be ubiquitous in nature.[19]–[21] Tβ4 functions mainly as an actin-sequestering molecule regulating cell migration, proliferation and differentiation.[22]–[25] It also promotes wound healing and modulates inflammatory mediators.[26], [27] Tβ4 was recently shown to promote cardiomyocyte migration, survival and repair in a coronary ligation model.[28]. Based on these mechanisms, we studied the effects of chronic Tβ4 administration on skeletal and cardiac muscle function in exercised dystrophin deficient mice. While we found no significant differences in muscle function, we did see significantly increased skeletal muscle regeneration in Tβ4 treated *mdx* mice and these regenerating fibers distinctly stained for Tβ4.

## Methods

### Animal Care

All animals were handled in strict accordance with good animal practice as defined by the relevant national and/or local animal welfare bodies, and all animal work was approved by the Institutional Animal Care and Use Committee at the Children's National Medical Center, Washington, DC and the Veterans Administration Medical Center, Washington, DC (Protocol #01079). Eight to ten week old female C57BL/10ScSn-Dmd*^mdx^*/J (*mdx*) and C57BL/10ScSnJ (wild type) mice weighing 20–30 grams were purchased from The Jackson Laboratory (Bar Harbor, Ma). All mice were housed in an individually vented cage system with a 12 hour light-dark cycle and received standard mouse chow and water *ad libitum*. All mice were rested at least 10–14 days before starting acclimations and baseline recordings.

### Treatment with Thymosin Beta-4

Tβ4 (RegeneRx Biopharmaceuticals Inc., Bethesda, Md) was given via intraperitoneal injection twice weekly over a 6 month period to *mdx* and wild type mice at a dose of 150 µg in 200 µl PBS. Buffer was given at the same times to *mdx* and wild type control groups.

### Treadmill Exercise

The treadmill exercise uses a common commercially available setup (Columbus Instruments, Columbus, Ohio) which employs a moving belt. We subjected all experimental mice to a 30-minute run on a horizontal treadmill at 12 m/min, twice a week. This test was performed during the morning hours twice weekly during the 6 months except in those days on which functional data was obtained.

### Grip Strength Test

Grip Strength was assessed using a grip strength meter consisting of horizontal forelimb mesh and an angled hind limb mesh (Columbus Instruments, Columbus, OH). Five successful hindlimb and forelimb strength measurements within 2 minutes were recorded and normalized to body weight as previously described.[29]

### Rotarod Test

Mice were trained on the Rotarod (Ugo Basile, VA, Italy) for two days before collecting data. Each acclimatization session consisted of four training sessions, 2 per day and each session lasting 120 seconds at a speed of 5 rpm. Each trial consisted of placing the mice on the rod at 10 rpm for 60 seconds (stabilizing period) followed by an acceleration from 10 rpm to 40 rpm within the first 25 seconds until the animal falls from the rod or until 180 seconds are reached. If the animals fell during the stabilizing period, they were placed back on the rod to complete the session. The total testing time is 240 seconds (60 sec stabilization time and 180 seconds test time). Each trial was done twice a day (a gap of 2 h interval between sessions) for 3 consecutive days. The latency to fall (seconds) was recorded and all six scores per mouse were averaged and recorded as latency to fall (in seconds) for each mouse.

### Echocardiography

Mice were anesthetized with 1–2% isoflurane in 100% oxygen and scanning was performed over 20 minutes using a high frequency ultrasound probe (RMZ 702a, Vevo 660, VisualSonics, Toronto, Canada) as previously described.[8] Qualitative and quantitative measurements were made offline using analytic software (VisualSonics, Toronto, Canada).

### Histological Evaluations

At the end of the trial, all animals were euthanized and tissue samples were taken for further testing. Histological evaluations were done by two individuals in a blinded manner using coded H&E stained slides and their results were averaged. The number of tissues examined per group varied based on tissue availability. Quantitative stereology (Olympus C.A.S.T. Stereology System, Olympus America Inc., Center Valley, PA) was used to evaluate the slides. Assessment criteria included: assessment of total fibers present, total fibers with central nuclei, total peripheral nuclei, total central nuclei, regenerating fibers (highly basophilic fibers), degenerating fibers, and inflammation (an interstitial group of more than 10 smaller inflammatory cell dark blue nuclei in a high power field) in five high power (40x) non-overlapping fields in normal and *mdx* gastrocnemius muscle sections. Fibers intersecting the left and top borders of the field where not counted and nuclei further than one nuclear diameter from the fiber border were deemed “central”.[29]

For immunohistochemistry, tissue slides of untreated mdx gastrocnemius skeletal muscle were deparaffinized and hydrated. For antigen retrieval, slides were immersed in citrate buffer (0.01 M, pH 6.0) and heated twice in a microwave (700 W or high) for 5 min. The slides were quenched with endogenous peroxidase by incubation with 3% hydrogen peroxide solution for 5 minutes and washed three times in PBS for 5 minutes. Slides were then immunostained with rabbit polyclonal antibody to thymosin β4 (1∶2000 dilution; ALPCO Diagnostics, Windham, NH, USA) at 4°C overnight. After primary antibody incubation, slides were washed three times in PBS for 5 minutes and incubated with secondary antibody for 1 hour. Then, slides were washed four times in PBS for 5 minutes each and the color reaction was developed with DAB and slides were counterstained Meyer's hematoxylin (DAKO, Carpinteria, CA, USA) for 20 seconds, dehydrated, and mounted with Permount (Fisher Scientific, Pittsburgh, PA, USA).

### Quantification of Fibrosis

Using gastrocnemius, diaphragm and cardiac muscle tissue from treated and untreated wild type and *mdx* mice, five paraffin embedded sections for each group were stained with hematoxylin and eosin (H&E) (Sigma, St. Louis, Mo) and Gomori's Tri-Chrome stain containing: fast green FCF, chromotrope 2R, and phosphotungstic acid (Sigma, St. Louis, Mo). The tissue was imaged under a light microscope at 10X and digital images were obtained using computer software (Olympus C.A.S.T. Stereology System, Olympus America Inc., Center Valley, PA). The digital images were copied into NIH Image J program and threshold set to separate blue staining collagen from red staining muscle tissues. The total area of blue staining collagen was then expressed as a percent of total tissue area in the image.

### Creatine Kinase (CK) Determination

Blood was obtained by heart puncture immediately after euthanasia. 250 µL of blood was collected into eppendorf tubes, allowed to clot and kept at room temperature to allow clot-contraction prior to centrifugation and serum collection. CK determination was performed according to the manufacturer's instructions using standard spectrophotometric method with enzyme-coupled assay reagent from Fisher Scientific (CK10).[30] Absorption at 340 nm was measured every min for 2 min at 37°C to calculate enzyme activity. Duplicate measurements were done on each serum sample and the data was expressed as U/L.

### Statistical Analysis

Measurements between wild type and *mdx* mice were compared at each time point using an analysis of variance with Sidak adjustment for multiple comparisons (body weight, GSM, Rotarod, echocardiography, percent collagen). Normality of each quantitative measurement was confirmed prior to analysis and those not conforming to normality underwent data transformations. Histology measurements (degeneration fibers, regenerating fibers, inflammation, calcification, and central and peripheral nuclei) were first compared between two investigators to determine their consistency. Comparisons were then made using Poisson regression or using negative binomial regression where the Poisson model did not fit the data due to over dispersion.

## Results

**Figure 1:**
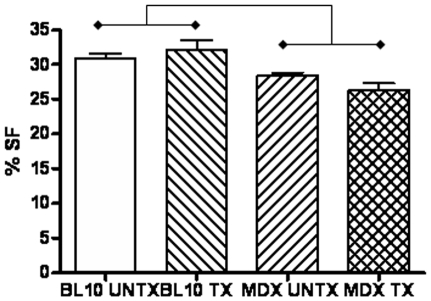
Significantly decreased cardiac function (mean ± SEM) measured as percent shortening fraction (%SF) in *mdx* mice is seen after 6 months of treatment with thymosin-beta 4 compared to wild type mice. There is no significant difference between treated and untreated *mdx* mice.

**Figure 2:**
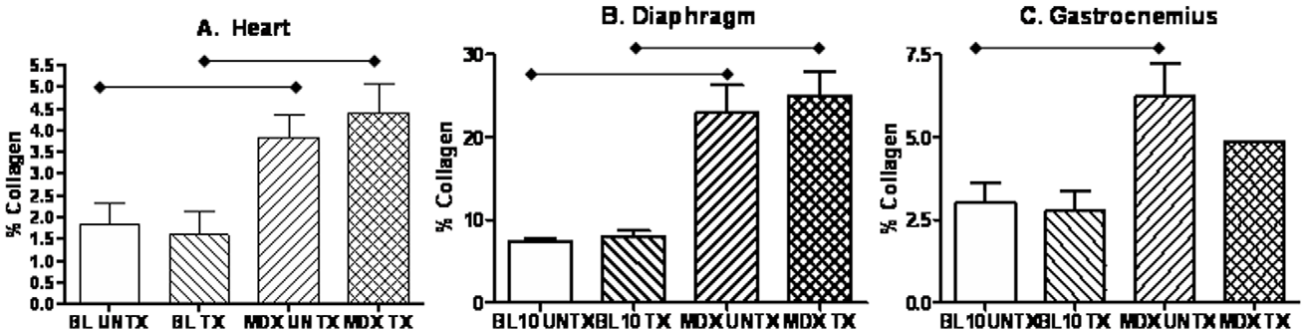
Significantly increased percent collagen (mean ± SEM) for cardiac (n = 3 for treated and untreated *mdx* mice and untreated wild type, n = 4 for treated wild type; panel A), diaphragm (n = 5 for all groups; panel B), and gastrocnemius (n = 3 for all groups; panel C) is seen in *mdx* mice compared to wild type. There was no significant difference between treated and untreated *mdx* mice.

Both wild type and *mdx* mice were treated with Tβ4 for 6 months and the behavioral data was collected at baseline (3 months of age), mid trial (5–6 months of age), and end of the trial (9 months of age). All results are presented as mean ± standard deviation except those in figures 1 and 2.

### Body Weight

No significant differences were seen in body weights between treated and untreated mice within the same strain. When comparing *mdx* and wild type mice, there was a significant increase in the body weight of *mdx* mice at baseline and 6 months but not at 9 months of age (Table S1).

### Grip Strength

Untreated *mdx* mice had significantly decreased normalized forelimb grip strength (kilogram force per kilogram; KGF/kg) compared to untreated wild type mice at 3, 6 and 9 months of age. Comparing the normalized hindlimb grip strength of these same groups showed a significant decrease in *mdx* mice at 3 and 6 months but not at 9 months of age. There were no significant differences in normalized forelimb or hindlimb grip strength between treated and untreated mice within the same strain during the trial (Table S1).

### Rotarod

There were no significant differences in latency time to fall on the Rotarod apparatus between wild type and *mdx* mice. There were also no differences between treated and untreated mice within the strain, but treated *mdx* mice showed significantly lower performance in comparison with treated wild type mice at 9 months of age (Table S1).

### Echocardiography

High frequency echocardiography found decreased cardiac function, measured as percent shortening fraction, in untreated *mdx* (27.9±1.86%) mice compared to untreated (30.6±2.6%) and treated (32.0±5.2; p = 0.045) wild type mice (Figure 1). Treated *mdx* (26.2±3.1%) mice also showed significantly decreased cardiac function compared to treated (p<0.01) and untreated (p<0.05) wild type mice. There were no significant differences between treated and untreated mice within the same strain. No significant differences were found in measurements of left ventricular chamber size or wall thickness between *mdx* and wild type mice, showing no dilation in the hearts of *mdx* mice with decreased function. No significant differences were found in heart rates or Doppler measurements of aortic, pulmonary, tricuspid or mitral blood flow velocities between treated and untreated *mdx* mice (Table S2).

### Skeletal Muscle Histology

Evaluation of the gastrocnemius skeletal muscle histology found significantly increased number of regenerating fibers in treated *mdx* mice (11.6±13.5) compared to untreated *mdx* mice (2.6±1.1; p = 0.03). Both treated and untreated mdx mice had increased central nuclei, central nuclei per fiber and central nucleated fibers compared to treated and untreated wild type. Untreated *mdx* mice showed significantly increased total peripheral nuclei compared to treated *mdx* mice (p = 0.014). There was also significantly increased inflammation (3+) between *mdx* and wild type mice that was not significantly altered in the treated groups (Table S3).

### Quantification of Fibrosis

**Figure 3:**
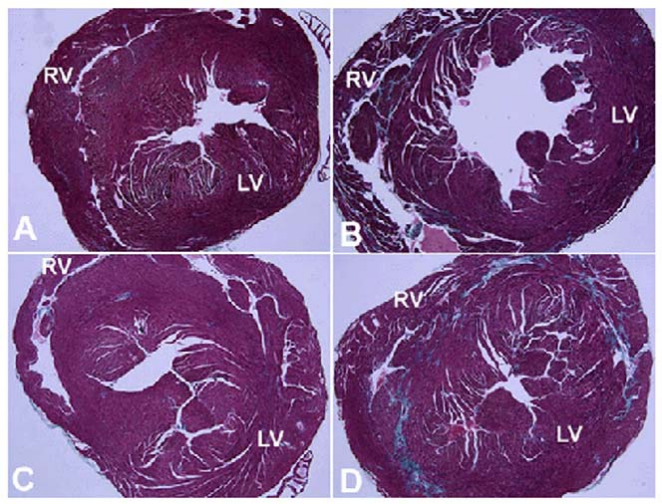
Gomori's tri-chrome stained slides of cardiac tissue showing increased fibrosis in *mdx* mice. A) Untreated wild type cardiac tissue showing minimal collagen staining (light blue color) corresponding to a percent collagen of 1.82±0.5%. B) Untreated *mdx* cardiac tissue showing diffuse fibrosis in the LV and RV ventricular walls corresponding to a percent collagen of 3.83±0.5%. C) Tβ4 treated wild type mice showing few increased areas of collagen staining corresponding to a percent collagen of 1.6±0.5%. D) Tβ4 treated *mdx* cardiac tissue showing large areas of collagen staining in the LV and RV walls corresponding to a percent collagen of 4.39±0.7%. (LV – left ventricle, RV – right ventricle).

Using Gomori's tri-chrome staining, an analysis of percent collagen showed significantly increased collagen found in the left ventricles of untreated (3.83±0.9%) and treated (4.39±1.2%) *mdx* mice compared to both untreated (1.6±1.1%) and treated (1.82±0.9%) wild type controls (all p values<0.05) (Figures 2 and 3). The diaphragm also showed significantly increased percent collagen in the untreated (22.9±7.5%) and treated (25.0±6.6%) *mdx* mice compared to untreated (7.4±0.8%) and treated (7.9±1.8%) control mice (all p values<0.01). The gastrocnemius also showed significantly increased percent collagen in untreated *mdx* mice (6.25±1.7%) compared to untreated control mice (3.0±1.1%; p<0.05) (Figure 2). There were no significant differences in percent collagen between treated and untreated groups within the same strain.

### Serum Creatine Kinase

There was a significant increase in serum creatine kinase in both treated (5205±1785 U/L, n = 8) and untreated (5788±2494 U/L, n = 10) *mdx* mice compared to treated (141±108 U/L, n = 14) and untreated (85±75 U/L, n = 13) wild type controls (p<0.001). There was no significant difference between treated and untreated *mdx* mice.

### Tβ4 Localization Using Immunohistochemistry

**Figure 4:**
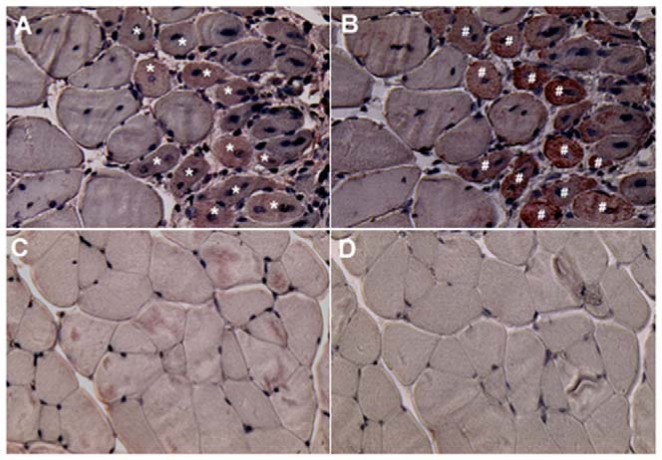
Peroxidase staining of regenerating fibers using anti-TB4 antibodies in skeletal muscle (gastrocnemius). A) *Mdx* muscle treated with anti-TB4 antibody shows peroxidase staining of regenerating fibers (*); B) *Mdx* muscle treated with anti-desmin antibody shows staining in regenerating fibers (#), the same as in plate A; C) Wild type muscle treated with anti-TB4 antibodies shows no staining; D) Wild type muscle treated with anti-desmin antibodies shows no staining.

Staining of untreated wild type and mdx skeletal muscle (gastrocnemius) with anti-Tβ4 antibody shows localized staining in regenerating fibers. Sequential slides were stained for desmin, a marker for regenerating fibers, and this staining corresponded to Tβ4 staining. There was no staining of fibers with either anti-Tβ4 or anti-desmin antibodies in wild type tissue (Figure 4).

## Discussion

We completed a six month trial using Tβ4 in exercised *mdx* and wild type mice. We found no significant improvement in treated *mdx* skeletal or cardiac muscle function compared to untreated *mdx* mice. However, we did find significantly increased regenerating fibers in treated *mdx* skeletal muscle and these fibers convincingly stained for Tβ4. While Tβ4 led to increased regeneration in *mdx* skeletal muscle, it did not improve fibrosis in the cardiac, diaphragmatic or skeletal muscle of treated *mdx* mice. This study shows that chronic Tβ4 administration is beneficial for skeletal muscle fiber regeneration in dystrophin deficient mice.

Previous gene profiling experiments showed increased Tβ4 expression in skeletal muscle of *mdx* mice. Tseng et al. (2002) showed that in 16 week old *mdx* mice, the gastrocnemius muscle showed a two-fold increase in Tβ4 mRNA expression. Boer et al. (2002) showed that another member of the thymosin family with similar properties, thymosin beta-10, showed a 4-fold increased expression in 13–15 week old *mdx* gastrocnemius muscle compared to wild type.[9] Nakayama et al. (2004) found up-regulation of Tβ4 in 2 month old *mdx* hindlimb skeletal muscle cell culture and showed that it was not altered after the addition of micro-dystrophin to the culture.[11] However, the authors did not find a similar increase in DMD patient derived cell lines. Turk et al. (2005) found significantly increased prothymosin beta-4 (Ptmb4), the precursor protein, at 8 of 9 time points ranging from 1 to 20 weeks in *mdx* hindlimb muscle.[17] Hara et al. (2005) found up-regulation of Tβ4 expression in *mdx* skeletal muscle cell cultures and that Tβ4 stimulated migration and chemotaxis of myoblasts.[18] All of these studies used mice from 1 to 20 weeks old, a time period of rapid degeneration and regeneration in the *mdx* skeletal muscle and demonstrate that Tβ4 is important in skeletal muscle regenerative pathways. Our study supports these previous reports. We demonstrate the presence of Tβ4 in regenerating fibers (Figure 4) of *mdx* gastrocnemius muscle. We also showed a significantly increased number of regenerating fibers in the gastrocnemius of Tβ4 treated *mdx* mice (Table S3). This parameter has significant variation because the gastrocnemius muscle develops patchy areas of regeneration and the majority of the muscle that is sampled shows no areas of regeneration at all. In another study, Tβ4 stimulated the migration of stem cells in hair follicles leading to increased hair growth. [31] Tβ4 may likewise stimulate satellite cell migration in skeletal muscle cells, leading to improved regeneration. Importantly, this is the first correlation of gene expression data with *in vivo* administration and histological localization and supports an integral role for Tβ4 in muscle regeneration.

Another potential mechanism of Tβ4 mediated regeneration is the inhibition of apoptosis. Tβ4 was shown to decrease apoptosis in an ethanol-treated corneal epithelial model and inhibit activation of NF-kB during TNF-α stimulation in human corneal epithelial cells. [32], [33] In cardiac tissue, Bock-Marquette et al. (2004) showed that Tβ4 decreased cardiac fibrosis secondary to ischemic damage in a coronary ligation model. This beneficial effect of Tβ4 on myocyte cell survival was also related to decreased apoptosis and found to be mediated by PINCH, ILK and Akt. [28] Previous C2C12 muscle cell culture experiments from our lab also showed that Tβ4 directly decreased NF-kB activation from TNF-α stimulation. [34] These studies support the direct action of Tβ4 on muscle cells to inhibit NF-kB and consequently apoptosis and potentially improve muscle regenerative capacity.

Studies of Tβ4 in multiple tissue models showed modulation of various inflammatory cytokines. [35]–[37] While, these changes may acutely promote wound healing, the effects of chronic Tβ4 treatment on different cytokine levels are not known. Chronic treatment could induce a more prolonged cytokine response that may become more pro-inflammatory and pro-fibrotic, decreasing the beneficial effects seen with acute Tβ4 administration. This might explain why this study found no significant changes in the amounts of collagen in skeletal and cardiac muscle in chronically Tβ4 treated *mdx* mice.

Also, previous studies showed that decreased levels of Ac-SDKP, the active tetrapeptide that is released from Tβ4, led to increased cardiac and renal perivascular fibrosis.[38] Pokharel et al. (2004) also showed that in rats over-expressing angiotensin-converting enzyme, which decreases levels of Ac-SDKP, there was increased cardiac collagen content.[39] Thus, the chronic exposure of cardiac and skeletal muscle to Tβ4 may lead to a down-regulation of Tβ4 expression or receptor activity and a decrease in Ac-SDKP.

Although we did not directly measure Tβ4 levels in treated mice, a previous study showed significantly increased levels in the hearts and skeletal muscle of mice after treatment with 400 micrograms of Tβ4 via intraperitoneal injection.[40] Chronic exposure of Tβ4 could potentially lead to the development of anti-Tβ4 antibodies. These antibodies could neutralize Tβ4 and prevent any beneficial effects on cell survival and decreased fibrosis. The presence of any antibodies was not assessed in this study.

This study found a significant increase in Tβ4 positive regenerating fibers in the skeletal muscle of exercised *mdx* mice. There were no beneficial effects of chronic Tβ4 treatment on muscle function or fibrosis. This study provides histological correlation for previous gene expression studies showing the importance of Tβ4 in skeletal muscle regeneration.

## Supporting Information

Table S1 Body weight, normalized grip strength, and Rotarod latency to fall measurements in treated and untreated wild type (BL10) and mdx mice after 6 months of treatment with thymosin beta-4.
(0.05 MB DOC)

Table S2 Cardiac M-mode and spectral Doppler echocardiography measurements in treated and untreated wild type (BL10) and mdx mice after 6 months of treatment with thymosin beta-4.
(0.05 MB DOC)

Table S3 Gastrocnemius skeletal muscle histology measurements in treated and untreated wild type (BL10) and mdx mice after 6 months of treatment with thymosin beta-4.
(0.04 MB DOC)
