# Supplementary material for: Evaluation of Skeletal and Cardiac Muscle Function after Chronic Administration of Thymosin β-4 in the Dystrophin Deficient Mouse
Source: PLoS One. 2010 Jan 29;5(1):e8976. doi: 10.1371/journal.pone.0008976 (PMC2813286; doi:10.1371/journal.pone.0008976)
Supplement: Table S1 — Body weight, normalized grip strength, and Rotarod latency to fall measurements in treated and untreated wild type (BL10) and mdx mice after 6 months of treatment with thymosin beta-4. (0.05 MB DOC) [file pone.0008976.s001.doc]

Evaluation of Skeletal and Cardiac Muscle Function After Chronic Administration of Thymosin beta-4 in the Dystrophin Deficient Mouse

Supplemental Tables:

Table S1: Body weight, normalized grip strength, and Rotarod latency to fall measurements in treated and untreated wild type (BL10) and *mdx* mice after 6 months of treatment with thymosin beta-4.^1^

| **Parameter** | **Age** | **BL10 Treated (N=15)** | **BL10 Untreated (N=15)** | **MDX Treated (N=15)** | **MDX Untreated (N=13)** | **P-values for significantly different groups** |
| --- | --- | --- | --- | --- | --- | --- |
|  |  | **Mean ± SD** | **Mean ± SD** | **Mean ± SD** | **Mean ± SD** |  |
| Weight (g) | 3 months | 21.9 ± 1.3^a, b^ | 22.2 ± 0.9^c.d^ | 23.6 ±1.2^a,c^ | 23.6 ± 1.3^b, d^ | ^a^ (p=0.0018)  ^b^ (p=0.0024)  ^c^ (p=0.0108)  ^d^ (p=0.0131) |
| GSM forelimb (KGF/kg) |  | 5.6 ± 0.3^a, b^ | 5.5 ± 0.3^c, d^ | 4.6 ± 0.4^a, c^ | 4.4 ± 0.3^b, d^ | ^a^ (p<1.0e-5)  ^b^ (p<1.0e-5)  ^c^ (p<1.0e-5)  ^d^ (p<1.0e-5) |
| GSM hindlimb (KGF/kg) |  | 7.5 ± 0.5^a b^ | 7.3 ± 0.4^c, d^ | 6.3 ± 0.4^a, c^ | 6.2 ± 0.5^b, d^ | ^a^ (p<1.0e-5)  ^b^ (p<1.0e-5)  ^c^ (p<1.0e-5)  ^d^ (p<1.0e-5) |
| Latency to fall (s)*, ** |  | 14.84 ± 0.90 | 14.33 ± 1.47 | 14.28 ± 1.35 | 14.71 ± 1.39 | NS |
| Weight (g) ^ | 5-6 months | 21.8 ± 1.3^a, b^ | 22.1 ± 1.0^c, d^ | 24.3 ± 1.3^a, c^ | 24.9 ± 0.9^b, d^ | ^a^ (p<1.0e-5)  ^b^ (p<1.0e-5)  ^c^ (p=9.8e-5)  ^d^ (p<1.0e-5) |
| GSM forelimb (KGF/kg) ^ |  | 5.7 ± 0.5^a, b^ | 6.1 ± 0.5^c, d^ | 3.8 ± 0.4^a, c^ | 4.1 ± 0.4^b, d^ | ^a^ (p<1.0e-5)  ^b^ (p<1.0e-5)  ^c^ (p<1.0e-5)  ^d^ (p<1.0e-5) |
| GSM hindlimb (KGF/kg) ^ |  | 9.2 ± 0.7^a, b^ | 9.0 ± 0.6^c, d^ | 7.3 ± 0.7^a, c^ | 7.5 ± 0.6^b, d^ | ^a^ (p<1.0e-5)  ^b^ (p<1.0e-5)  ^c^ (p<1.0e-5)  ^d^ (p<1.0e-5) |
| Latency to fall (s)*, ^^ |  | 13.7 ± 1.8 | 12.9 ± 1.8 | 12.0 ± 2.0 | 13.0 ± 2.0 | NS |
| Weight (g) # | 9 months | 25.8 ± 1.9 | 26.9 ± 2.1 | 26.1 ± 0.8 | 27.3 ± 1.5 | NS |
| GSM forelimb (KGF/kg) # |  | 5.3 ± 0.6^a ,b^ | 5.4 ± 0.7^c, d^ | 4.0 ± 0.3^a, c^ | 4.1 ± 0.5^b, d^ | ^a^ (p<1.0e-5)  ^b^ (p=1.7e-5)  ^c^ (p<1.0e-5)  ^d^ (p<1.0e-5) |
| GSM hindlimb (KGF/kg) # |  | 9.1 ± 0.8 | 9.2 ± 1.2 | 8.7 ± 0.7 | 8.5 ± 1.1 | NS |
| Latency to fall (s)*, # |  | 13.7 ± 2.0^a^ | 12.5 ± 1.4 | 10.4 ± 2.0^a^ | 12.4 ± 2.6 | ^a^ (p=0.0036) |

NS – non-significant

* Latency to fall square root transformed due to lack of normality

** N=14 for MDX treated at 3 months

^ N=13 for MDX treated at 5-6 months; N=9 for MDX untreated at 5-6 months

^^ N=11 for MDX untreated at 5-6 months

# N=14 for BL10 untreated at 9 months; N=9 for MDX treated at 9 months; N=10 for MDX untreated at 9 months

^1^Portions of the data from untreated wild type and mdx mice were previously published. [29]
