# Supplementary material for: Evaluation of Skeletal and Cardiac Muscle Function after Chronic Administration of Thymosin β-4 in the Dystrophin Deficient Mouse
Source: PLoS One. 2010 Jan 29;5(1):e8976. doi: 10.1371/journal.pone.0008976 (PMC2813286; doi:10.1371/journal.pone.0008976)
Supplement: Table S2 — Cardiac M-mode and spectral Doppler echocardiography measurements in treated and untreated wild type (BL10) and mdx mice after 6 months of treatment with thymosin beta-4. (0.05 MB DOC) [file pone.0008976.s002.doc]

Evaluation of Skeletal and Cardiac Muscle Function After Chronic Administration of Thymosin beta-4 in the Dystrophin Deficient Mouse

Supplemental Tables:

Table S2: Cardiac M-mode and spectral Doppler echocardiography measurements in treated and untreated wild type (BL10) and *mdx* mice after 6 months of treatment with thymosin beta-4.^1^

| **Parameter** | **BL10 Treated (N=14)** | **BL10 Untreated (N=14)** | **MDX Treated (N=8)** | **MDX Untreated (N=10)** | **Overall p-value** | **P-values for significantly different groups** |
| --- | --- | --- | --- | --- | --- | --- |
|  | **Mean ± SD** | **Mean ± SD** | **Mean ± SD** | **Mean ± SD** |  |  |
| IVS (dia) (mm) | 0.83 ± 0.13 | 0.77 ± 0.13 | 0.88 ± 0.15 | 0.84 ± 0.16 | 0.3937 |  |
| IVS (sys) (mm) | 1.19 ± 0.15 | 1.10 ± 0.18 | 1.16 ± 0.19 | 1.12 ± 0.19 | 0.5274 |  |
| LVID (dia) (mm) | 3.67 ± 0.23^a^ | 3.90 ± 0.14^a^ | 3.79 ± 0.25 | 3.70 ± 0.19 | 0.0125 | ^a^ p=0.0249 |
| LVID (sys) (mm) | 2.50 ± 0.30^a^ | 2.73 ± 0.19 | 2.80 ± 0.27^a^ | 2.62 ± 0.11 | 0.0180 | ^a^ p=0.0337 |
| LV PW (dia) (mm) | 0.70 ± 0.11 | 0.70 ± 0.08 | 0.79 ± 0.11 | 0.71 ± 0.08 | 0.1475 |  |
| LV PW (sys) (mm)* | 0.007 ± 0.167 | -0.021 ± 0.090 | 0.061 ± 0.099 | -0.023 ± 0.103 | 0.4334 |  |
| LV mass (mg) | 82.3 ± 9.1 | 81.4 ± 8.7 | 84.7 ± 7.9 | 86.7 ± 7.1 | 0.4347 |  |
| Heart rate (bpm) | 421.5 ± 54.1^a^ | 408.4 ± 31.2^b^ | 454.6 ± 56.1 | 481.4 ± 43.5^a, b^ | 0.0021 | ^a^ p=0.0190  ^b^ p=0.0024 |
| Ao Vmax (mm/s) | 889.8 ± 109.6 | 866.5 ± 123.7 | 783.8 ± 107.6 | 813.0 ± 118.8 | 0.1525 |  |
| Ao VTI (mm) | 3.07 ± 0.43^a, b^ | 3.04 ± 0.51^c, d^ | 2.47 ± 0.48^a, c^ | 2.52 ± 0.26^b, d^ | 0.0018 | ^a^ p=0.0208  ^b^ p=0.0267  ^c^ p=0.0308  ^d^ p=0.0401 |
| PA Vmax (mm/s) | 615.9 ± 32.7 | 631.6 ± 41.5 | 615.5 ± 54.5 | 604.3 ± 48.0 | 0.4868 |  |
| PA VTI (mm) | 2.40 ± 0.18 | 2.51 ± 0.29^a^ | 2.19 ± 0.20^a^ | 2.28 ± 0.15 | 0.0089 | ^a^ p=0.0108 |
| MV e wave (mm/s) | 612.8 ± 45.2 | 620.9 ± 26.0 | 607.7 ± 23.2 | 622.0 ± 40.1 | 0.7806 |  |
| TV a wave (mm/s)* | 5.31 ± 0.15^a^ | 5.31 ± 0.14^b^ | 5.59 ± 0.31^a, b^ | 5.46 ± 0.31 | 0.0179 | ^a^ p=0.0424  ^b^ p=0.0372 |
| LV - %FS** | 32.04 ± 5.20^a,b^ | 30.63 ± 2.58^c^ | 26.23 ± 3.10^a,c^ | 27.89 ± 1.86^b^ | 0.0030 | ^a^ p=0.0042  ^b^ p=0.0447  ^c^p=0.0476 |

* Data log transformed to conform to normality

** BL10 treated group N=13

^1^Portions of the data from untreated wild type and mdx mice were previously published. [29]

IVS – interventricular septum, LVID – left ventricular internal diameter, LV PW – left ventricular posterior wall, LV – left ventricular, (dia) – diastole, (sys) – systole, Ao – aorta, PA – pulmonary artery, MV – mitral valve, TV – tricuspid valve, VTI – velocity time integral, Vmax – maximum velocity, bpm – beats per minute
