# Supplementary material for: Evaluation of Skeletal and Cardiac Muscle Function after Chronic Administration of Thymosin β-4 in the Dystrophin Deficient Mouse
Source: PLoS One. 2010 Jan 29;5(1):e8976. doi: 10.1371/journal.pone.0008976 (PMC2813286; doi:10.1371/journal.pone.0008976)
Supplement: Table S3 — Gastrocnemius skeletal muscle histology measurements in treated and untreated wild type (BL10) and mdx mice after 6 months of treatment with thymosin beta-4. (0.04 MB DOC) [file pone.0008976.s003.doc]

Evaluation of Skeletal and Cardiac Muscle Function After Chronic Administration of Thymosin beta-4 in the Dystrophin Deficient Mouse

Supplemental Tables:

Table S3: Gastrocnemius skeletal muscle histology measurements in treated and untreated wild type (BL10) and *mdx* mice after 6 months of treatment with thymosin beta-4.1

| **Parameter** | **BL10 Treated (N=7)** | **BL10 Untreated (N=7)** | **MDX Treated (N=7)** | **MDX Untreated (N=7)** | **P-values for significantly different groups** |
| --- | --- | --- | --- | --- | --- |
|  | **Mean ± SD** | **Mean ± SD** | **Mean ± SD** | **Mean ± SD** |  |
| Total fibers* | 77.9 ± 16.2^a^ | 76.8 ± 15.3^b^ | 93.5 ± 16.6 | 96.3 ± 22.6^a, b^ | ^a^ (p=0.039)  ^b^ (p=0.028) |
| Central nuclei* | 0.31 ± 0.30^a, b^ | 0.54 ± 0.36^c^ | 51.51 ± 12.00^a, c^ | 59.61 ± 15.31^b, c^ | ^a^ (p<1.0e-6)  ^b^ (p<1.0e-6)  ^c^ (p<1.0e-6)  ^d^ (p<1.0e-6) |
| Peripheral nuclei* | 88.7 ± 18.66 | 101.4 ± 18.21 | 82.3 ± 21.99^a^ | 107.9 ± 26.35^a^ | ^a^ (p=0.0139) |
| Central nucleated fibers* | 0.31 ± 0.30^a, b^ | 0.51 ± 0.38^c, d^ | 39.88 ± 9.13^a, c^ | 43.05 ± 10.97^b, d^ | ^a^ (p<1.0e-6)  ^b^ (p<1.0e-6)  ^c^ (p<1.0e-6)  ^d^ (p<1.0e-6) |
| Peripheral nuclei per fiber^ | 1.15 ± 0.14^a^ | 1.33 ± 0.11^b^ | 0.92 ± 0.17^a, b^ | 1.13 ± 0.15 | ^a^ (p=0.0459)  ^b^ (p=2.5e-5) |
| Central nuclei per fiber**, ^ | 3.4e-5 ± 4.8e-5^a, b^ | 7.1e-5 ± 8.7e-5^c, d^ | 0.3 ± 0.07^a, c^ | 0.4 ± 0.19^b, d^ | ^a^ (p<1.0e-6)  ^b^ (p<1.0e-6)  ^c^ (p<1.0e-6)  ^d^ (p<1.0e-6) |
| Degenerating fibers*, ^^ | 3.6 ± 5.9 | 0.0 ± 0.0 | 6.0 ± 6.1 | 3.9 ± 4.9 |  |
| Regenerating fibers*, ^^ | 0.8 ± 2.1 | 0.0 ± 0.0^a^ | 11.6 ± 13.5^a, b^ | 2.6 ± 1.1^b^ | ^a^ (p=2.1e-5)  ^b^ (p=0.0332) |
| Inflammation*, ^^ | 0.7 ± 1.6^a, b^ | 0.3 ± 0.7^c, d^ | 5.8 ± 3.4^a, c^ | 7.0 ± 5.0^b, d^ | ^a^ (p=3.0e-6)  ^b^ (p<1.0e-6)  ^c^ (p<1.0e-6)  ^d^ (P<1.0e-6) |

* Analyzed using negative binomial regression for count data

**Data square transformed to conform to normality

^ Analyzed using ANOVA

^^ Sample size: N=15 for BL10 treated, N=14 for BL10 untreated, N=9 for MDX treated and N=7 for MDX untreated

^1^Portions of the data from untreated wild type and mdx mice were previously published. [29]
